# Isolated Rare Urethral Metastasis From Primary Lung Adenocarcinoma: Case Report and Literature Review

**DOI:** 10.3389/fonc.2019.00784

**Published:** 2019-08-20

**Authors:** Xin Zhu, Heqiu Li, Shuang Li, Mi Zhou

**Affiliations:** ^1^Department of Urology, The First Affiliated Hospital of Chongqing Medical University, Chongqing, China; ^2^Department of Pathology, Molecular Medicine Testing Center, Chongqing Medical University, Chongqing, China; ^3^Department of General Surgery, Jinshan Branch, The First Affiliated Hospital of Chongqing Medical University, Chongqing, China; ^4^Department of Respiratory and Critical Care Medicine, The First Affiliated Hospital of Chongqing Medical University, Chongqing, China

**Keywords:** lung adenocarcinoma, metastasis, case report, literature review, urethra

## Abstract

**Background:** Lung adenocarcinoma is a common respiratory malignancy, however urethral metastasis of lung adenocarcinoma has not yet been reported. This study aims to present a rare case of isolated urethral metastasis in a male patient with a history of primary lung adenocarcinoma.

**Case Presentation:** A 69 year-old male patient was admitted complaining of dysuria and nocturia for 3 months, with a history of lung adenocarcinoma after surgery. The patient was diagnosed as benign prostatic hyperplasia (BPH) and received holmium laser enucleation of the prostate, an effective transurethral procedure to treat bladder outflow obstruction due to BPH. Four months after surgery for BPH, the patient had no improvement in symptoms and continued to complain of dysuria and perineum pain. An MRI of the pelvis indicated posterior urethral mass without any regional lymphadenopathy or other sites of lesion. Urethrocystoscopy found the mass in the membranous urethra near the verumontanum, and pathology combined with immunohistochemical staining confirmed the isolated urethral metastasis of lung adenocarcinoma. The further therapeutic regimen consisting of chemotherapy (pemetrexed combined with nedaplatin) and bevacizumab was well-tolerated, and obviously relieved the patient from dysuria and perineum pain.

**Conclusion:** This study reported the first case of isolated rare urethral metastasis from primary lung adenocarcinoma and underlined the necessity for clinicians to remain vigilant to metastasis during follow-up of primary cancer.

## Introduction

Lung cancer represents the most common cancer, with 228,150 estimated new cases and 142,670 estimated deaths in the United States in 2019 (1). Lung cancer is categorized into small cell lung cancer (SCLC), accounting for 15% of cases, and non-small cell lung cancer (NSCLC), around 50% of which are adenocarcinoma histology (2, 3). Surgical resection still remains the single most successful option to cure patients with lung cancer, however metastasis of primary cancer exerts as a significant factor affecting cancer-specific survival. The most common metastatic sites of lung cancer include the nervous system, bone, liver, respiratory system and adrenal gland (4, 5). sHowever, the metastasis to urinary system is rare, to our knowledge, this is the first report of isolated urethral metastasis from lung adenocarcinoma.

## Case Presentation

**Figure 1:**
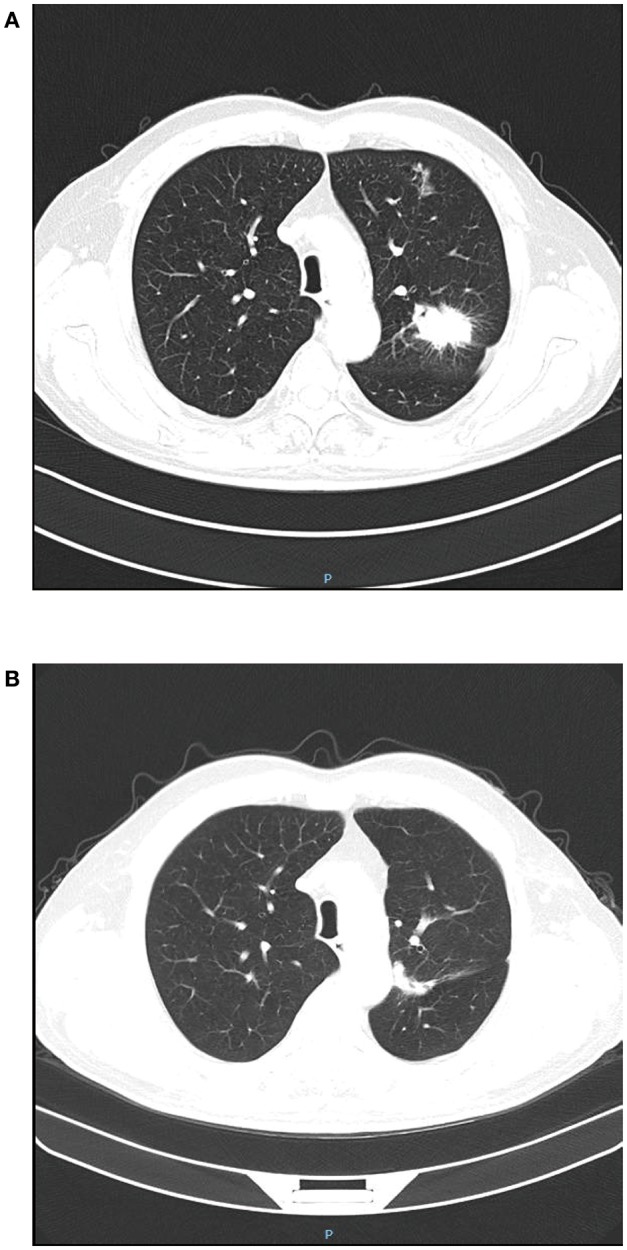
Chest CT before and after video-assisted wedge resection of left upper lung lobe. **(A)** The lung tumor located in the left upper lung lobe; **(B)** Fourteen months after resection of lung tumor in the left upper lung lobe.

**Figure 2:**
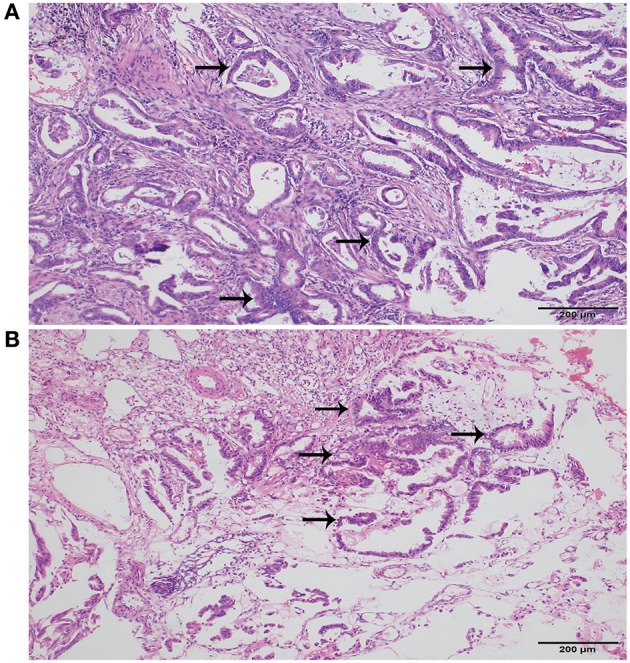
Histopathology of lung and urethra cancer. **(A)** Primary adenocarcinoma of lung (Hematoxylin and Eosin, original magnification × 100). **(B)** Metastatic urethral adenocarcinoma (Hematoxylin and Eosin, original magnification × 100). Black arrows indicated adenocarcinoma.

**Figure 3:**
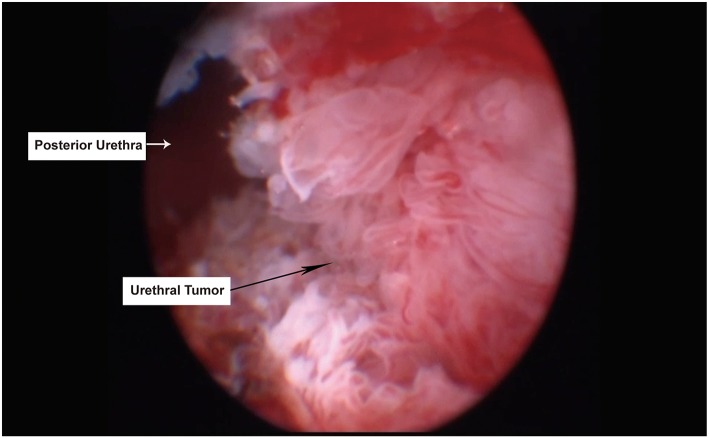
Tumor located in the membranous urethra under transurethral endoscopy. The black arrow indicated urethral tumor, while white arrow indicated normal posterior urethra.

A 69 year-old male patient was admitted with difficulty urinating and nocturia for 3 months. The digital rectal examination did not indicate any hardness or nodules of the prostate, and an abdominal ultrasound revealed an enlarged prostate and the prostate specific antigen (PSA) value was normal. Fourteen months prior to this admission, he had undergone video-assisted thoracic surgery for the wedge resection of left upper lung lobe due to the left upper lung mass found in chest CT scan (Figure 1A), and pathology revealed infiltrating lung adenocarcinoma with T_2_N_0_M_0_ staging (Figure 2A). Re-examination of chest CT on this admission indicated no evidence of disease recurrence after lung lesion resection (Figure 1B). Then the patient was diagnosed with benign prostatic hyperplasia (BPH) and received holmium laser enucleation of the prostate, an effective transurethral procedure to treat bladder outflow obstruction due to BPH. The pathology revealed benign prostate hyperplasia as expected. Four months after surgery for BPH, the patient had no improvement in symptoms and continued to complain of dysuria and perineum pain. Urinalysis was negative for infection and PSA was not elevated, however an MRI of the pelvis indicated posterior urethral mass without any regional lymphadenopathy or other sites of lesion. Urethrocystoscopy under general anesthesia found the mass in the membranous urethra near the verumontanum (Figure 3), and transurethral loop electrosurgical excision was conducted to remove the mass. Due to the infiltration of tumor into normal urethral tissue, the transurethral operation aimed to resect as much tumor tissue as possible, so as to facilitate pathological diagnosis, but it was difficult to guarantee negative margins. Routine hematoxylin and eosin stained sections indicated adenocarcinoma (Figure 2B) and further immunohistochemical staining showed negative PSA, positive thyroid transcription factor 1 (TTF1) and Napsin A, confirming the urethral metastasis of lung adenocarcinoma. To identify the latent metastases to other sites and recurrence from primary lung adenocarcinoma, the patient underwent brain MRI, chest CT, abdominal ultrasound and emission computed tomography for whole-body bone scan and received negative results, suggesting isolated urethral metastases. The patient was restaged as T2N0M1 of lung adenocarcinoma, and chose 6 courses of systematic chemotherapy (pemetrexed combined with nedaplatin) plus bevacizumab, a monoclonal antibody targeting vascular endothelial growth factor at 3 weeks intervals. The therapeutic regimen was well-tolerated, and obviously relieved the patient from dysuria and perineum pain.

## Discussion

Lung cancer is the most common malignancy threatening human health, and metastasis of lung cancer is a significant prognosis-associated factor for patients. Previous studies have shown that the most common metastatic sites in lung cancer are nervous system, bone, liver, respiratory system and adrenal gland (4, 5). A retrospective study from a single clinical center found that the uncommon metastases from NSCLC included kidney, pancreas, spleen, peritoneum, intestine, bone marrow, eye, ovary, thyroid, heart, breast, tonsil, and nasal cavity, which were also identified as predictors for poor prognosis (6). The identity of metastasis usually comes from routine follow-up examination or specific symptoms caused by metastatic lesions. With consideration to curative treatment for lung cancer, the early detection and instant treatment for silent metastasis to brain, bone, and liver, the most common metastatic sites, conferred benefits to quality of life and survival (7). With regard to uncommon metastatic sites, choroidal metastasis was reported in several case reports and detected on clinical characteristics of reduction in visual acuity and blurred vision (8, 9). Cases of gastrointestinal metastasis from lung cancer have been occasionally detected during staging work-up or based on abdominal symptoms, including gastric metastasis (10, 11), small intestine (12), and colorectal metastasis (13, 14). Hu et al. performed a retrospective analysis of 366 gastrointestinal metastases cases from lung cancer and found that the small intestine and colorectum were the two most common metastatic organs of lung cancer in the gastrointestinal tract.

Metastasis from lung cancer to urinary system is rare and the majority of the reported cases focus on kidney and bladder. Verma et al. reported that stereotactic body radiation therapy exerted as a safe and effective treatment to palliatively relieve renal metastases from non-small cell lung cancer (15). Bladder metastasis from lung adenocarcinoma was also reported in some reports, with clinical symptoms of hematuria (16, 17). The epidemiological information about metastatic urethral cancer was scarce and reported in limited case reports (18), and the bulbar urethra is the most encountered section of the male urethra, among which acute urinary retention, lower urinary tract symptoms, and hematuria are the main symptoms. Although Tefilli et al. reported the urethral metastasis from lung carcinoma, however the case had been diagnosed as lung giant cell carcinoma with brain metastases previously (19).

There are currently no standard treatments for isolated urethral metastases given the rarity of this condition. Based on the guideline of primary urethral cancer, in locally advanced urethral carcinoma, cisplatin-based chemotherapy with curative intent prior to surgery was beneficial for survival compared to chemotherapy alone, or surgery followed by chemotherapy (20). In this case, the main lesion was located in the membranous urethra near the verumontanum, and the further treatment was selected as cisplatin-based chemotherapy and bevacizumab. Compared with the chemotherapy-alone group, the addition of bevacizumab to chemotherapy improved overall survival and progression-free survival for advanced non-squamous non-small-cell lung cancer (21). With regards to chemotherapy, combination of pemetrexed and cisplatin was recommended as the first-line option for advanced NSCLC (22, 23). In this case, chemotherapy (pemetrexed and nedaplatin) plus bevacizumab were effective to relieve local symptoms with good tolerability. Moreover, increasing evidence supported to combine immunotherapy using checkpoint inhibitors with platinum-based chemotherapy as first-line treatment in advanced NSCLC, with improved response rates and survival (24, 25). For oligometastatic lesion, local therapies including surgery, radiation therapy, radiofrequency ablation, or cryoablation palliated symptoms in patients with advanced NSCLC and potentially avoid switching systemic therapy prematurely (26, 27).

In this report, we present the first isolated urethra metastasis from lung adenocarcinoma. This rare case could not only add new evidence for the unreported metastatic sites of lung adenocarcinoma in the literature, but also provide lessons for clinicians. The patient's chief complaint of dysuria was routinely considered to be associated with BPH and an unnecessary surgery for BPH was performed. Although ultrasound of prostate indicated enlarged prostate and pathology also confirmed the diagnosis of BPH, however BPH was not the main reason for dysuria. For a male lung cancer patient, especially when the duration of lower urinary symptoms was short, clinicians should consider the possibility of urethral metastasis in the differential diagnosis for persistent dysuria after appropriate treatment for BPH.

## Author Contributions

XZ: data collection and manuscript writing. HL: data collection. SL: data collection. MZ: project development, data collection, and manuscript writing.

### Conflict of Interest Statement

The authors declare that the research was conducted in the absence of any commercial or financial relationships that could be construed as a potential conflict of interest.

## References

[1] Siegel RL Miller KD Jemal A Cancer statistics, 2019. CA Cancer J Clin. (2019) 69:7–34. 10.3322/caac.21551 30620402

[2] Sher T Dy GK Adjei AA. Small cell lung cancer. Mayo Clin Proc. (2008) 83:355–67. 10.4065/83.3.355 18316005

[3] Denisenko TV Budkevich IN Zhivotovsky B. Cell death-based treatment of lung adenocarcinoma. Cell Death Dis. (2018) 9:117. 10.1038/s41419-017-0063-y 29371589 PMC5833343

[4] Quint LE Tummala S Brisson LJ Francis IR Krupnick AS Kazerooni EA. Distribution of distant metastases from newly diagnosed non-small cell lung cancer. Ann Thorac Surg. (1996) 62:246–50. 10.1016/0003-4975(96)00220-2 8678651

[5] Riihimaki M Hemminki A Fallah M Thomsen H Sundquist K Sundquist J. Metastatic sites and survival in lung cancer. Lung Cancer. (2014) 86:78–84. 10.1016/j.lungcan.2014.07.020 25130083

[6] Niu FY Zhou Q Yang JJ Zhong WZ Chen ZH Deng W. Distribution and prognosis of uncommon metastases from non-small cell lung cancer. BMC Cancer. (2016) 16:149. 10.1186/s12885-016-2169-5 26911831 PMC4766662

[7] Sanchez de Cos J Sojo Gonzalez MA Montero MV Perez Calvo MC Vicente MJ Valle MH. Non-small cell lung cancer and silent brain metastasis. Survival and prognostic factors. Lung Cancer. (2009) 63:140–5. 10.1016/j.lungcan.2008.04.013 18556086

[8] Asteriou C Konstantinou D Kleontas A Paliouras D Samanidis G Papadopoulou F. Blurred vision due to choroidal metastasis as the first manifestation of lung cancer: a case report. World J Surg Oncol. (2010) 8:2. 10.1186/1477-7819-8-2 20064203 PMC2817644

[9] Funazo T Morita K Ikegami N Konishi C Nakao S Ariyasu R. Successful treatment with alectinib for choroidal metastasis in anaplastic lymphoma kinase rearranged non-small cell lung cancer. Intern Med. (2017) 56:2317–20. 10.2169/internalmedicine.8488-16 28794371 PMC5635306

[10] Gao S Hu XD Wang SZ Liu N Zhao W Yu QX. Gastric metastasis from small cell lung cancer: a case report. World J Gastroenterol. (2015) 21:1684–8. 10.3748/wjg.v21.i5.1684 25663792 PMC4316115

[11] Sharma P Dwary AD Khan EM. Serendipitous discovery of isolated gastric metastases from adenocarcinoma of the lung on staging 18F-FDG PET-CT. Clin Nucl Med. (2017) 42:807–8. 10.1097/RLU.0000000000001784 28806259

[12] Akamatsu H Tsuya A Kaira K Nakamura Y Naito T Murakami H Intestinal metastasis from non-small-cell lung cancer initially detected by (1)(8)F-fluorodeoxyglucose positron emission tomography. Jpn J Radiol. (2010) 28:684–7. 10.1007/s11604-010-0470-9 21113753

[13] Han SH Lee JW Hyun CL Yoo SY Lee JH Kwon JM. Solitary rectal metastasis from primary small cell lung carcinoma. Thorac Cancer. (2012) 3:284–6. 10.1111/j.1759-7714.2011.00091.x 28920312

[14] Sakai H Egi H Hinoi T Tokunaga M Kawaguchi Y Shinomura M. Primary lung cancer presenting with metastasis to the colon: a case report. World J Surg Oncol. (2012) 10:127. 10.1186/1477-7819-10-127 22741562 PMC3433337

[15] Verma V Simone CB II. Stereotactic body radiation therapy for metastases to the kidney in patients with non-small cell lung cancer: a new treatment paradigm for durable palliation. Ann Palliat Med. (2017) 6:96–103. 10.21037/apm.2017.03.06 28480719

[16] Cormio L Sanguedolce F Di Fino G Massenio P Liuzzi G Bufo P. Bladder metastasis from lung adenocarcinoma: a difficult differential diagnosis with primary bladder adenocarcinoma. World J Surg Oncol. (2014) 12:90. 10.1186/1477-7819-12-90 24716732 PMC3984282

[17] Raymond KW Hin TS Fai KC Steve CW. Urinary bladder metastasis from lung adenocarcinoma: a rare cause of hematuria. Urol Ann. (2014) 6:359–62. 10.4103/0974-7796.141006 25371617 PMC4216546

[18] Kazama S Kitayama J Sunami E Niimi A Nomiya A Homma Y. Urethral metastasis from a sigmoid colon carcinoma: a quite rare case report and review of the literature. BMC Surg. (2014) 14:31. 10.1186/1471-2482-14-31 24884559 PMC4037280

[19] Tefilli MV Stefani SD Mariano MB. Urethral metastasis of lung carcinoma with germinative cell features. Int Braz J Urol. (2003) 29:431–3. 10.1590/S1677-55382003000500008 15745589

[20] EAU Guidelines Presented at the EAU Annual Congress Barcelona 2019. (2019) Arnhem: EAU Guidelines Office Available online at: http://uroweb.org/guidelines/compilations-of-all-guidelines/

[21] Sandler A Gray R Perry MC Brahmer J Schiller JH Dowlati A Paclitaxel-carboplatin alone or with bevacizumab for non-small-cell lung cancer. N Engl J Med. (2006) 355:2542–50. 10.1056/NEJMoa061884 17167137

[22] Barlesi F Gervais R Lena H Hureaux J Berard H Paillotin D. Pemetrexed and cisplatin as first-line chemotherapy for advanced non-small-cell lung cancer (NSCLC) with asymptomatic inoperable brain metastases: a multicenter phase II trial (GFPC 07-01). Ann Oncol. (2011) 22:2466–70. 10.1093/annonc/mdr003 21321089

[23] He G Xiao X Zou M Zhang C Xia S. Pemetrexed/cisplatin as first-line chemotherapy for advanced lung cancer with brain metastases: a case report and literature review. Medicine. (2016) 95:e4401. 10.1097/MD.0000000000004401 27512852 PMC4985307

[24] Ellis PM Vella ET Ung YC. Immune checkpoint inhibitors for patients with advanced non-small-cell lung cancer: a systematic review. Clin Lung Cancer. (2017) 18:444–59.e1. 10.1016/j.cllc.2017.02.001 28416123

[25] Pacheco JM Camidge DR Doebele RC Schenk E. A changing of the guard: immune checkpoint inhibitors with and without chemotherapy as first line treatment for metastatic non-small cell lung cancer. Front Oncol. (2019) 9:195. 10.3389/fonc.2019.00195 30984621 PMC6450209

[26] Gomez DR Blumenschein GR Jr Lee JJ Hernandez M Ye R Camidge DR. Local consolidative therapy versus maintenance therapy or observation for patients with oligometastatic non-small-cell lung cancer without progression after first-line systemic therapy: a multicentre, randomised, controlled, phase 2 study. Lancet Oncol. (2016) 17:1672–82. 10.1016/S1470-2045(16)30532-0 27789196 PMC5143183

[27] Kim C Hoang CD Kesarwala AH Schrump DS Guha U Rajan A. Role of local ablative therapy in patients with oligometastatic and oligoprogressive non-small cell lung cancer. J Thorac Oncol. (2017) 12:179–93. 10.1016/j.jtho.2016.10.012 27780780

